# Adult Plant Slow Rusting Genes Confer High Levels of Resistance to Rusts in Bread Wheat Cultivars From Mexico

**DOI:** 10.3389/fpls.2020.00824

**Published:** 2020-07-14

**Authors:** Julio Huerta-Espino, Ravi Singh, Leonardo A. Crespo-Herrera, Héctor E. Villaseñor-Mir, Maria F. Rodriguez-Garcia, Susanne Dreisigacker, Daniel Barcenas-Santana, Evans Lagudah

**Affiliations:** ^1^Campo Experimental Valle de México INIFAP, Chapingo, Edo. de México, Mexico; ^2^Global Wheat Program, International Maize and Wheat Improvement Center (CIMMYT), México City, Mexico; ^3^Instituto Tecnológico Superior de Tlatlauquitepec, Puebla, Mexico; ^4^CSIRO Agriculture and Food, Canberra, ACT, Australia

**Keywords:** wheat, resistance, slow rusting, genes, molecular markers

## Abstract

Rust diseases continuously threaten global wheat production: stem rust, leaf rust, and yellow rust caused by *Puccinia graminis* f. sp. *tritici*, *Puccinia triticina*, and *Puccinia striiformis* f. sp. *tritici*, respectively. Recent studies indicated that the average losses from all these three rusts reached up to 15.04 million tons per year, which is equivalent to an annual average loss of around US $2.9 billion per year. The major focus of Mexican and worldwide breeding programs is the release of rust resistant cultivars, as this is considered the best option for controlling rust diseases. In Mexico, the emphasis has been placed on genes that confer partial resistance in the adult plant stage and against a broad spectrum of rust races since the 1970s. In this study, a set of the first-generation tall varieties developed and released in the 1940s and 1950s, the first semi-dwarfs, and other releases in Mexico, all of which showed different levels of rust resistance have been phenotyped for the three rust diseases and genotyped. Results of the molecular marker detection indicated that *Lr34*, *Lr46*, *Lr67*, and *Lr68* alone or in different gene combinations were present among the wheat cultivars. Flag leaf tip necrosis was present in all cultivars and most were positive for brown necrosis or Pseudo Black Chaff associated with the *Sr2* stem rust resistance complex. The phenotypic responses to the different rust infections indicate the presence of additional slow rusting and race-specific resistance genes. The study reveals the association of the slow rusting genes with durable resistance to the three rusts including Ug99 in cultivars bred before the green revolution such as Frontera, Supremo 211, Chapingo 48, Yaqui 50, Kentana 52, Bajio 52, Bajio 53, Yaqui 53, Chapingo 53, Yaktana Tardio 54, and Mayo 54 and their descendants after intercrossing and recombination. These slow rusting genes are the backbone of the resistance in the current Mexican germplasm.

## Introduction

The three rust diseases continuously threaten global wheat production: stem rust, leaf rust, and yellow rust caused by *Puccinia graminis* f. sp. *tritici* (Pgt), *Puccinia triticina* (Pt), and *Puccinia striiformis* f.sp. *tritici* (Pst), respectively. It is estimated that global annual losses to wheat rust pathogens range between US$ 4.3 to 5.0 billion (Philp Pardey University of Minnesota, unpublished; Figueroa et al., 2018). The deployment of rust resistant cultivars is considered the best option to control rust diseases and their development is the major focus of the breeding program at CIMMYT and worldwide.

Resistance to wheat rusts is generally categorized into two non-exclusive types, race-specific and race non-specific. Race-specific resistance is generally qualitative and usually short-lived due to the evolution of potentially virulent pathogens (Huerta-Espino et al., 2011; Wellings, 2011). In contrast, adequate levels of race non-specific resistance involve genes which might contribute from minor to intermediate effects. Plants carrying this type of resistance are susceptible at the seedling stage but express resistance at the post-seedling stages of plant growth. This characteristic is called slow rusting and often associated with some forms of adult plant resistance (APR) (Lagudah, 2011).

In Mexico, emphasis has been placed on genes that confer partial resistance in the adult plant stage since the 1950s. The most important genes belonging to the category of “slow rusting” or APR include *Lr34/Yr18//Sr57/Ltn1* (Singh et al., 2012), *Lr46/Yr29//Sr58/Ltn2* (Singh et al., 2013) and *Lr67/Yr46//Sr55/Ltn3* (Herrera-Foessel et al., 2014), which confer partial resistance to leaf rust, yellow rust, and stem rust. These genes are associated with flag leaf tip necrosis (LTN) a post-flowering morphological trait (William et al., 2003; Singh et al., 2011; Herrera-Foessel et al., 2014). Another important gene in this category is *Lr68/Ltn4*, which confers resistance to leaf rust (Herrera-Foessel et al., 2012), and apparently confers a certain degree of stem rust resistance not yet quantified and confirmed. Another example is the *Sr2* gene for resistance to stem rust which provides useful protection, although at a lower level, than most of the race specific stem rust genes. It confers an “adult plant” type of resistance and became the backbone of the Mexican germplasm being effective since their introduction until now (Singh et al., 2011; Singh et al., 2015). The *Sr2* gene expresses as a “slow rusting” type of resistance in which the rate of epidemic development is considerable reduced. *Sr2/Yr30* confers resistance to stem rust and yellow rust (Singh et al., 2005; Singh et al., 2008; Mago et al., 2011b). This gene is derived from the cultivar Hope and had provided durable resistance to stem rust in the CIMMYT-Mexican spring wheat germplasm and can be identified by its linkage with the pseudo-black chaff or brown necrosis phenotype observed on the glumes and below the nodes (Borlaug et al., 1949).

The effects of these APR genes when alone, are moderate, however, they play an important role in gene combinations and interactions with other major genes and a range of minor QTLs that cause additive effects, resulting in high levels of durable resistance. In general, accumulating APR genes into a single cultivar can result in “near-immunity'' or a high level of resistance: three to four in the case of leaf rust, four to five in the case of yellow rust (Singh et al., 2000), but more than five for stem rust (Knott, 1988; Singh et al., 2015).

The objective of the present study was to investigate the presence and effectiveness of the known/unknown slow rusting resistance genes against the three rust diseases in selected tall and dwarf wheat cultivars released in Mexico

## Materials and Methods

### Plant Material

Fifty-one bread wheat cultivars (**Table 1**) released in Mexico before and after the green revolution era were characterized in seedling tests in the greenhouse (GH) and in the field against stem rust, leaf rust, and yellow rust.

**Table 1:** Mexican bread wheat cultivars, their parents, and year of release used in the study to determine the response to stem rust (SR), leaf rust (LR), and yellow rust (YR).

Variety	Year of release	Type	Cross
FRONTERA	1943	Tall	FRONTEIRA//HOPE/MEDITERRANEAN
SUPREMO 211	1943	Tall	SUPRESA//HOPE/MEDITERRANEAN
MARROQUI 588	1945	Tall	FLORENCE/AURORE
CANDEAL 48	1948	Tall	SELECCION CANDEAL
CHAPINGO 48	1948	Tall	NEWTHATCH/MARROQUI
KENTANA 48	1948	Tall	KENYA 324/MENTANA
YAQUI 48	1948	Tall	NEWTHATCH/MARROQUI
YAQUI 48	1948	Tall	NEWTHATCH/MARROQUI
EGIPTO 101	1950	Tall	KENYA GOVERNOR= K155
HUAMANTLA	1950	Tall	FROCOR//FRONTANA/YAQUI 50
LERMA	1950	Tall	MENTANA 3*/KENYA
LERMA 50	1950	Tall	MENTANA 3*/KENYA
LERMA ROJO	1950	Tall	LEE/FRONTANA//KENTANA 52A
NAYAR	1950	Tall	NEWTHATCH/AGUILERA 4//MTA/TH
VERANO PELON	1950	Tall	NEWTHATCH/AGUILERA 4//MTA/TH
YAQUI 50	1950	Tall	NEWTHATCH/MARROQUI
KENTANA 51	1951	Tall	KENYA 324/MENTANA
BAJIO 52	1952	Tall	YAQUI 48/KENTANA 48
CANDEAL 52	1952	Tall	NEWTHATCH/TORREON
KENTANA 52	1952	Tall	KENYA 324/MENTANA
YAKTANA TARDIO (YAKNA T)	1952	Tall	YAQUI 48/KENTANA 48//FRONTANA
BAJIO 53	1953	Tall	YAQUI 48/KENTANA 48
CHAPINGO 53	1953	Tall	KENTANA 48/YAQUI 48
CONSTITUCION	1953	Tall	MARROQUI 588/TH//EGYPT 101/GABO
TOLUCA 53	1953	Tall	YAQUI 48/KENTANA 48
YAQUI 53	1953	Tall	YAQUI 48//EGYPT 101/GABO
HUAMANTLA ROJO (HUA R)	1954	Tall	LEE/FRONTANA//KENTANA 52A
KENTANA 54	1954	Tall	KENTANA/RIO NEGRO
LERMA ROJO	1954	Tall	LERMA 50/3/Y48/2*ME//SPO211
MAYO 54	1954	Tall	EGYPT 101/GABO//MAYO 48
NARINO 59	1959	Tall	FROCOR/MCM//KT48/Y48
CRESPO 63	1963	Tall	FCR/4/N/2*/MTA//K/3/BAGE/5/GB54
LERMA ROJO 64	1964	Dwarf	Y50/N10b//l52/3/2*Lr
JARAL F66	1966	Dwarf	Sn64a//Tzpp/Nai60
TOBARI F66	1966	Dwarf	Tzzp/Sn64a
BAJIO M67	1967	Dwarf	Tzzp/Sn64a
NORTENO M67	1967	Dwarf	Lr64/Sn64
SARIC F70	1970	Dwarf	Cno//Sn64/Klre/3/8156
CHAPINGO VF74	1974	Dwarf	Fraternal Selection
ORIZABA 77	1977	Dwarf	CIANO F 67/SIETE CERROS T 66//CC/TOBARI F 66
YECORATO F77	1977	Dwarf	Inia66/Cno//Cal/3/Yr resel (b)
HUASTECO M81	1981	Dwarf	Hop/Ron//Kal
VICTORIA M81	1981	Dwarf	Yr resel(b)/Trf//Rsk/Trm
GUARINA 85	1985	Dwarf	TR791767(F5)/3/H/RA//2F2
CURINDA M87	1987	Dwarf	Ias58/4/Kal/Bb//Cj71/3/Ald
MOCHIS T88	1988	Dwarf	Prl/Vee#6
TEPOCA M89	1989	Dwarf	Buc/4/Tzpp//Irn46/Cno67/3/Prt
BATAN F92	1992	Dwarf	Cno67/Mfd//Moncho/Seri M92
HUITES F95	1995	Dwarf	Vee/Koel
PALMERIN F2005	2005	Dwarf	Borl95/Rabe
MAYA S2007	2007	Dwarf	845.63.6/SLM//CUBA/3/CALIOPA-E-B/4/LIMPIA

### Seedling Testing

#### Stem Rust

Seedling evaluations against the stem rust RTR race were conducted at CIMMYT's GH facilities in El Batan, Mexico. Eight to 10 seeds per cultivar were sown per entry in plastic trays, as well as a set of both 20 differential from the Minnesota Cereal Disease Laboratory (CDL) (Jin et al., 2007) and the CIMMYT standard Pgt set (Singh, 1991). The seedling plants were inoculated 10 days after planting, when the seedlings had developed the second leaf, using urediniospore sprays of the RTR Pgt race (**Table 2**) suspended in Soltrol 170^®^ (Phillips Petroleum Company, Borger, TX) mineral oil (Singh, 1991) at a concentration of 5 mg/ml (Herrera-Foessel et al., 2007). Plants were placed inside a dew chamber overnight and then moved back to the GH.

**Table 2:** Avirulence and virulence status of stem rust, stripe rust, and leaf rust and pathotypes used to inoculate at seedling in the greenhouse and adult plant in the field the 51 cultivars of bread wheat included in the study.

Rust	Race	Avirulence genes	Virulence genes	Reference
*Stem rust*				
	RTR	*Sr7a*,*9e*,*10*,*12*,*13*,*14*,*22*,*23*,*24*,*25*,*26*,*27*,*29*,*30*,*31*,*32*, *33*,*35*, *Dp2*, *H*	*Sr5*,*6*,*7b*,*8a*,*8b*,*9a*,*9b*,*9d*,*9f*,*9g*,*11*,*15*,*17*,*21*,*28*,*34*,*36*	Singh, 1991
	TTKSK	*Sr24*,*36*, *Tmp*	*Sr5*,*6*,*7b*, *8a*, *9a*,*9b*,*9d*,*9e*,*9g*,*10*,*11*,*17*,*30*,*31*,*38*, *McN*	Jin et al., 2007
*Stripe rust*				
	Mex14.191	*Yr1*,*4*,*5a*,*10*,*15*,*24*, *26*,*5b*, *Poll*	*Yr2*,*3*,*6*,*7*,*8*,*9*,*17*,*27*,*31*, *A*	Randhawa et al., 2018
*Leaf rust*				
	MBJ/SP	*Lr2a*,*2b*,*2c*,*3ka*,*9*,*16*,*19*,*21*,*24*,*25*,*28*,*29*,*30*,*32*,*33*,*36*	*Lr1*,*3*,*3bg*,*10*,*11*,*12*,*13*,*14a*,*14b*,*15*,*17a*,*18*,*20*,*23*, *(26)*,*27+31*	Herrera-Foessel et al., 2012

A second set of the 51 cultivars and susceptible checks were tested against Pgt race TTKSK at the seedling stage at the United States Department of Agriculture - Agricultural Research Service (USDA-ARS) CDL, St. Paul, MN, following the procedure described by Rouse et al. (2011) and Jin et al. (2007). Ten seeds of each cultivar were planted in trays. Eight-day-old seedlings were inoculated with fresh urediniospores of Pgt race TTKSK.

In both SR evaluations, infection types (ITs) on seedlings were recorded 14 days post-inoculation using a 0 to 4 scale as described by Stakman et al. (1962). ITs “0”, “1”, and “2” were considered resistant, whereas ITs “3” to “4” were considered susceptible.

#### Leaf Rust

Seedling tests were carried out at CIMMYT headquarters GH. Single spore races were used in the evaluations. Wheat seedlings were grown for 10 days under GH conditions (20°C/23°C night/day temperatures) and inoculated with the rust in a Soltrol 170 spore suspension at a concentration of 5 mg/ml. The LR race used at seedling test was MBJ/SP described by Herrera-Foessel et al. (2012) (**Table 2**). After the inoculation, plants were kept in a humid chamber at 22°C for 16 h. Plants were then placed back in the GH for 2 more weeks and scored for IT on a 0–4 scale as described in Roelfs et al. (1992).

#### Yellow Rust

Nine to 12-day-old seedlings (two-leaf stage) were inoculated by using an atomizer to spray urediniospores suspended in lightweight mineral oil, Soltrol 170 at a concentration of 5 mg/ml. The yellow rust race used in the study was MEX14.191 (Randhawa et al., 2018). The trays carrying inoculated seedlings were then placed in a mist chamber and incubated at 7–9°C for 24 h. Seedlings were then moved to a GH room maintained at 15/18°C night/day temperatures. The ITs were recorded 14 days post-inoculation using a 0–9 scale (McNeal et al., 1971).

### Field Testing

The 51 bread wheat cultivars and the susceptible checks as follows: Sr RTR-Apav 1; TTKSK (UG99) Cacuke; Lr Avocet and Apav 1; Yr Avocet and Apav 1 were evaluated in field experiments conducted at the CIMMYT-CENEB (Campo Experimental Normal E. Borlaug) in Cd. Obregon, Sonora and at El Batan (BV) research stations in Mexico. Cultivars were evaluated at CIMMYT-CENEB for stem rust during 2014–15, 2015–16, and 2016–17 and against LR during the 2014–15, 2015–16, and during summer 2016 at El Batan. The cultivars were evaluated against yellow rust at the CIMMYT-Toluca (MV) experimental station during 2014, 2015, 2016, and at BV during 2016. All the cultivars were also evaluated during 2016 at the off and main growing seasons for SR (Ug99) at Kenya Agricultural Research Institute (KARI).

In all locations and years, about 5-g seed of each cultivar was sown in 0.7-m long paired-row raised-bed plots with a spacing of 0.3 m between them in Mexico and on flat beds in Njoro, Kenya, where one row was left unplanted between plots.

Spreader rows were planted on each side of the experimental area, as well as hill plots in the middle of the 30-cm pathway to the side of each experimental plot. The spreaders consisted of a mixture of susceptible cultivars (differing) for each rust disease at Cd. Obregon (Mexico). For the stem rust TTKSK race a mixture of the stem rust susceptible wheat cultivar Cacuke and six Sr24-carrying cultivars were used. For leaf rust, a mixture of Avocet resistant to yellow rust was used, whereas in the case of YR, a mixture of wheat cultivars carrying *Yr9*+*Yr27*, Morocco, and Avocet near-isogeneic cultivars for gene *Yr17* and *Yr31* was used as spreader.

### Spreader Inoculation

The stem rust and leaf rust spreaders at Cd. Obregon were inoculated with spores of the Mexican Pgt race RTR and Pt race MBJ/SP (Singh, 1991), respectively, suspended in Soltrol 170 at a concentration of 5 mg/ml 8 weeks after sowing. In Njoro, the spreaders were inoculated with a field collection of stem rust races including TTKSK by spraying with a mixture of urediniospores suspended in water at a concentration of 5 mg/ml plus Tween 20 and performing needle inoculations using the same suspension (Jin et al., 2008).

For the yellow rust evaluations, the Mexican Pst race Mex14.191 suspended in Soltrol 170 at a concentration of 5 mg/ml was inoculated into YR spreaders (Ponce-Molina et al., 2018). The inoculation method was as described by Lan et al. (2014).

### Rust Evaluation

Disease severity (DS) on the cultivars was scored at two to three times in each experiment using the modified Cobb's scale (Peterson et al., 1948) and host response to infection was determined according to Roelfs et al. (1992).

Cultivars were further categorized based on the final DS (Singh et al., 2008; Huerta-Espino et al., 2013) as following: near-immune (NI, 1% severity), resistant (R, 5–10% severity), resistant to moderately resistant (RMR, 15–20% severity), moderately resistant (MR, 30% severity), moderately resistant to moderately susceptible (MRMS, 40% severity), moderately susceptible (MS, 50–60% severity), moderately susceptible to susceptible (MSS, 70–80% severity), and susceptible (S, 90–100% severity) compared to the necrotic status, due to 100% DS, for the susceptible checks as follows: stem rust RTR-Apav 1; TTKSK (UG99) Cacuke; leaf rust Avocet and Apav 1; for yellow rust Avocet and Apav 1.

### Leaf Tip Necrosis (LTN) and Pseudo Black Chaff (PBC) or Brown Necrosis

LTN and PBC morphological traits were scored as “+” when visible at all locations and years; if not clear or the presence of PBC was in doubt, we did not give the “+” score.

### Genotyping Slow Rusting Genes *via* Molecular Markers

Gene-based and closely linked molecular markers were used to determine the presence of the resistance alleles of genes *Lr34*, *Lr46*, *Lr67*, *Lr68*, and *Sr2* (**Supplemental Table 2**). For *Lr34*, a SNP marker designed around the 3bp indel in exon 11 was used (Lagudah et al., 2009). The *Lr46* gene is not cloned yet, therefore functional markers were not available. Two SNP markers in the proximity of *Lr46* were therefore deployed (Viccars, L., Chandramohan, S., and Lagudah, E. unpublished data). For *Lr67*, three diagnostic markers were applied, one sequence tag site (STS marker) and two SNP markers. Markers included the functional SNP observed in exon 2 of the gene (SNP_TM4) (Moore et al., 2015). A SNP marker derived from the linked CAPS marker, cs7BLNLRR, was used to determine the resistance allele for the gene *Lr68*. As with *Lr46*, *Lr68*, and *Sr2* have not yet been cloned and hence no functional marker at present exist. To infer the resistance allele for *Sr2*, a SNP marker based on linked CAPS marker csSr2 was applied (Mago et al., 2011a). In addition to gene-based markers for slow rusting resistance, markers for the two most important height reducing genes (*Rht-B1* and *Rht-D1*) were evaluated.

The SNP polymorphisms were scored using Kompetitive Allele Specific PCR (KASP) reagents (www.lgcgenomics.com) in reactions containing 2.5-ml water, 2.5-ml 2×KASPar Reaction mix, 0.07-ml assay mix and 50 ng of dried DNA with a polymerase chain reaction (PCR) profile of 94°C for 15 min activation time followed by 20 cycles of 94°C for 10 s, 57°C for 5 s, and 72°C for 10 s which were then followed by 18 cycles of 94°C for 10 s, 57°C for 20 s, and 72°C for 40 s. Fluorescence was read as an end point reading at 25°C.

The PCR assay reaction mixture in single 10-µl reactions used to amplify the STS marker for *Lr67* contained final concentrations of 1× Buffer with Green Dye (Promega Corp., US), 200 µM deoxynucleotide triphosphates (dNTPs), 1.2 mM magnesium chloride (MgCl2), 0.25 µM of each primer, 1U of DNA polymerase (GoTaq^®^Flexi, Promega Corp., Cat. # M8295) and 50ng of DNA template. The PCR profile was 94°C for 2 min followed by 30 cycles of 94°C for 1 min, 56°C and 72°C for 2 min. The amplified products were separated on 1.2% agarose gels in tris-acetate/ethylene-diamine-tetra acetic acid (TAE) buffer.

## Results

Cultivars under evaluation were separated into two groups, tall and dwarf in order to see the possible negative or positive effect of the dwarfing genes on the disease resistance. The grouping allowed us to estimate the frequency, i.e., presence/absence of the slow rusting resistance genes before and after the dwarfing genes were introduced and other relatively recent released materials.

### Seedling Testing

At seedling stage, all tall cultivars tested (except Marroqui 588 and Lerma 50) were resistant to the RTR stem rust race; all dwarfs were also resistant to Pgt race RTR. Yaqui 50, Yaqui 53, Bajio 53, Chapingo 53, and Crespo 63 were resistant to the TTKSK (Ug99) stem rust race and the rest were susceptible. In the case of leaf rust, most cultivars tested were susceptible except Bajio 52, Toluca 53, and Bajio 53 among the tall (**Table 3**) and Curinda M86 and Mochis T88 among the dwarfs (**Table 4**). In the case of yellow rust, a behavior like that of leaf rust was observed, where most of the cultivars were susceptible as seedlings to race MEX14.191, except for Marroqui 588, Chapingo 48 and Kentana 48 among the tall, and Tobari F66, Orizaba 77, Huasteco M81, Huites F95, and Maya S2007 among the dwarfs.

**Table 3:** Year of release and response to stem rust, leaf rust, and yellow rust at seedling and maximum disease severity at the field evaluation of Mexican tall cultivars.

VARIETY	Year of Release	STEM RUST						LEAF RUST			YELLOW RUST		
		Seedling	Maximum disease	Resistance group	Seedling	Maximum disease	Resistance group	Seedling	Maximum disease	Resistance group	Seedling	Maximum disease	Resistance group
		RTR			TTKSK			MBJ/SP			MEX14.191		
SUPREMO 211	1945	2+	5	R	3	15	RMR	4	15	RMR	7	15	RMR
MARROQUI 588	1945	3+	40	MRMS	3+	50	MS	4	5	R	4	20	RMR
FRONTERA	1945	X	60	MS	3	60	MS	4	1	NI	7	20	RMR
CANDEAL 48	1948	2=	0	NI	3	30	MR	4	1	NI	7	20	RMR
CHAPINGO 48	1948	2C	10	R	3+	30	MR	4	10	R	3	5	R
YAQUI 48	1948	X-	20	RMR	3	20	RMR	4	70	MS	7	70	MSS
KENTANA 48	1948	2+	60	MS	3+	60	MS	4	5	R	5	10	R
VERANO PELON	1950	2	1	NI	3+	15	RMR	4	5	R	7	40	MRMS
EGYPT 101	1950	2	1	NI	3	20	RMR	4	1	NI	7	40	MRMS
YAQUI 50	1950	2C	5	R	2+	10	R	4	10	R	7	40	MRMS
YAQUI 50	1950	X-	5	R	3+	20	RMR	4	1	NI	7	40	MRMS
LERMA ROJO	1950	2	10	R	3+	60	MS	4	15	RMR	7	30	MS
LERMA	1950	;1	30	MR	3+	60	MS	4	5	R	7	50	MS
NAYAR	1950	2+C	40	MRMS	3	40	MRMS	3+	5	R	7	40	MRMS
HUAMANTLA	1950	;1=	40	MRMS	3+	50	MS	4	5	R	8	50	MS
LERMA 50	1950	3	70	MSS	3+	60	MS	4	5	R	7	15	RMR
KENTANA 51	1951	2	60	MS	3+	60	MS	4	1	NI	7	15	RMR
CANDEAL 52	1952	2-	0	NI	3+	40	MRMS	4	1	NI	7	15	RMR
YAKNA T	1952	;1	20	RMR	3+	40	MRMS	4	0	NI	8	40	MRMS
KENTANA 52	1952	2+	40	MRMS	3+	40	MRMS	4	0	NI	7	1	NI
BAJIO 52	1952	2C	40	MRMS	2+	40	MRMS	X	5	R	7	30	MS
YAQUI 53	1953	;1	10	R	2	10	R	4	10	R	7	15	RMR
CHAPINGO 53	1953	2C	10	R	3	30	MR	4	10	R	7	40	MRMS
CONSTITUCION	1953	;	10	R	3+	50	MS	4	20	RMR	8	50	MS
TOLUCA 53	1953	2C	20	RMR	3	5	R	;1=	1	NI	6	20	RMR
BAJIO 53	1953	X	20	RMR	3	15	RMR	X	0	NI	7	15	RMR
LERMA ROJO	1954	1	20	RMR	3+	30	MR	4	10	R	8	50	MS
MAYO 54	1954	2	30	MR	3	40	MRMS	4	10	R	7	20	RMR
HUA R	1954	;1	30	MR	3+	60	MS	4	5	R	7	70	MSS
KENTANA 54	1954	X+	60	MS	3+	60	MS	4	5	R	7	70	MSS
NARINO 59	1959	2	20	RMR	3	10	R	4	1	NI	5	10	R
CRESPO 63	1963	2	30	MR	1+	5	R	4	0	NI	7	10	R

**Table 4:** Year of release and response to stem rust, leaf rust, and yellow rust at seedling and maximum disease severity at the field evaluation of Mexican dwarf cultivars.

VARIETY	Year of Release	STEM RUST						LEAF RUST			STRIPE RUST			
		Seedling	Maximum disease	Resistance group	Seedling	Maximum disease	Resistance group	Seedling	Maximum disease	Resistance group	Seedling	Maximum disease	Resistance group	
		RTR			TTKSK			MBJ/SP			MEX14.191		
LERMA ROJO 64	1964	;	5	R	3+	60	MS	4	30	MR	7	50	MS	
TOBARI F66	1966	0;	10	R	3+	50	MS	4	5	R	5	15	RMR	
JARAL F66	1966	2C	40	MRMS	3	40	MRMS	4	5	R	7	20	RMR	
NORTENO M67	1967	;	10	R	3+	30	MR	4	15	RMR	7	30	MR	
BAJIO M67	1967	0;	10	R	3+	40	MRMS	4	15	RMR	7	30	MR	
SARIC F70	1970	1-	0	NI	3+	10	R	4	5	R	7	15	RMR	
CHAPINGO VF74	1974	;1	1	NI	3+	30	MR	4	40	MRMS	5	5	R	
ORIZABA 77	1977	;	15	RMR	3+	40	MRMS	4	5	R	0	1	NI	
YECORATO F77	1977	;	5	R	3+	70	MSS	4	20	RMR	7	40	MRMS	
VICTORIA M81	1981	1-	5	R	3+	30	MR	4	15	RMR	5	40	MRMS	
HUASTECO M81	1981	2=	20	RMR	3+	60	MS	4	20	RMR	7	50	MS	
GUARINA 85	1985	;	0	NI	3	5	R	4	5	R	5	20	RMR	
CURINDA M87	1987	1-	40	MRMS	3+	60	MS	X	15	RMR	8	60	MS	
MOCHIS T88	1988	;	20	RMR	3+	70	MSS	X	1	NI	8	60	MS	
TEPOCA M89	1989	;1-	10	R	3+	50	MS	4	5	R	7	60	MS	
BATAN F92	1992	;	10	R	3+	60	MS	4	20	RMR	7	40	MRMS	
HUITES F95	1995	;	5	R	3+	40	MRMS	4	20	RMR	5	10	R	
PALMERIN F2005	2005	;	0	NI	3+	50	MS	4	1	NI	7	5	R	
MAYA S2007	2007	0;	5	R	3+	10	R	4	15	RMR	4	5	R	

### Field Testing

**Figure 1:**
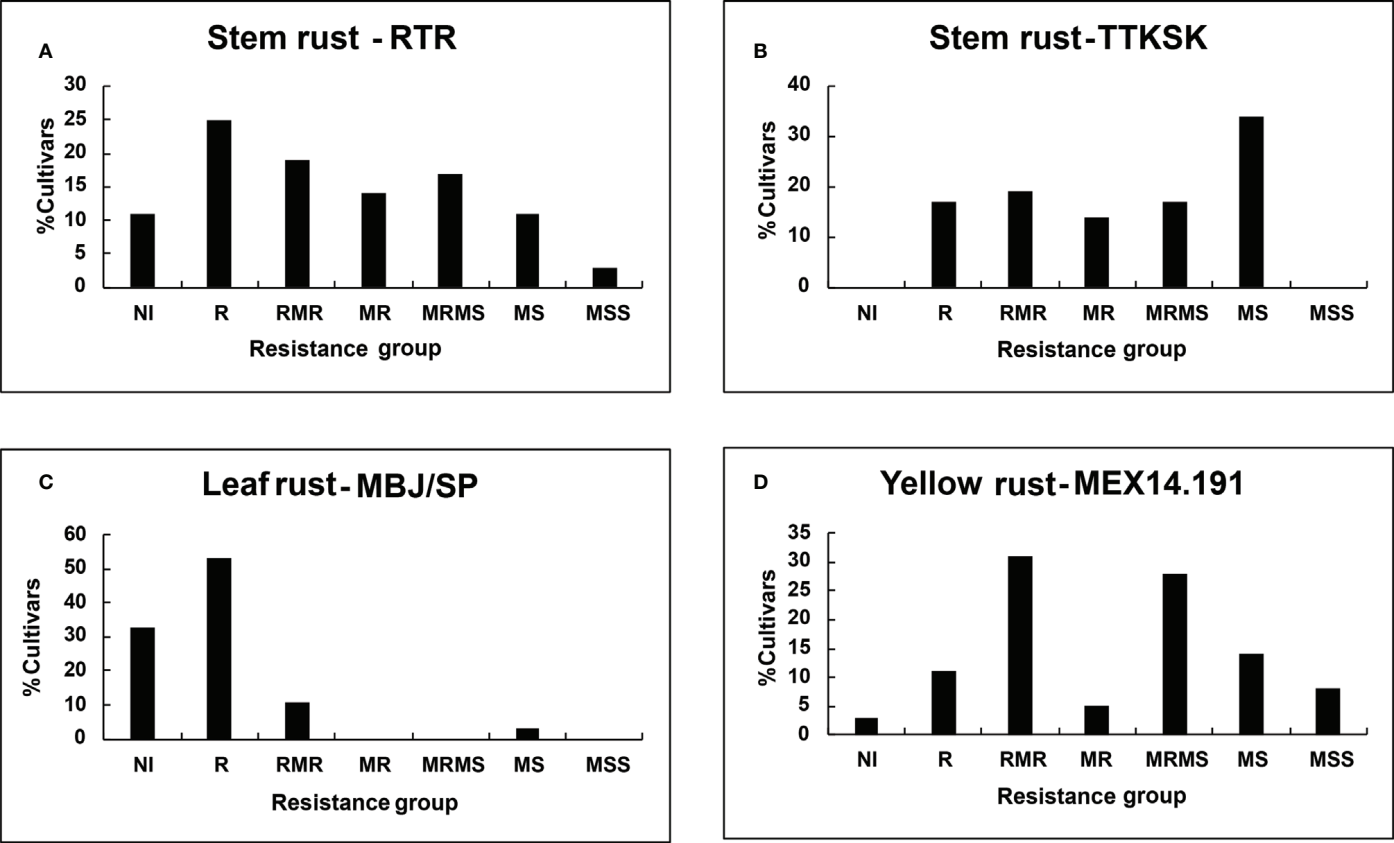
Percent of tall cultivars grouped on the basis of final disease severity: Stem rust races **(A)** RTR; **(B)** TTKSK; **(C)** Leaf rust race MBJ/SP; **(D)** Yellow rust race MEX14.191.

**Figure 2:**
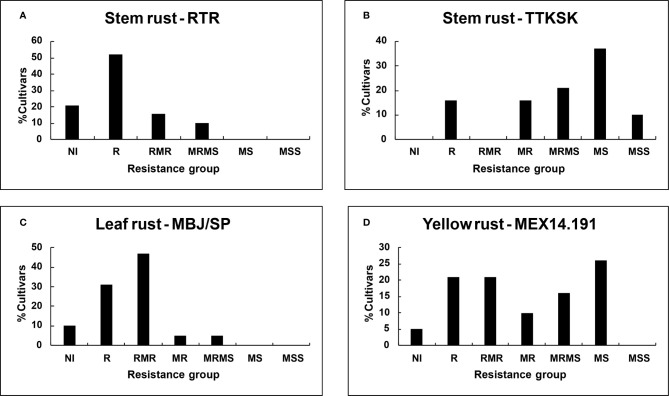
Percent of dwarf cultivars grouped on the basis of final disease severity: Stem rust races **(A)** RTR; **(B)** TTKSK; **(C)** Leaf rust race MBJ/SP; **(D)** Yellow rust race MEX14.191.

Despite seedling resistance against Pgt race RTR of stem rust, different degrees of final disease severities were observed among the tall cultivars. These responses varied from near immune (NI) response in Candeal 48, Candeal 52, Verano Pelon, and Egipto101, to MSS in Lerma 50; and from NI in Saric F70, Guarina 85, Palmerin F2005 and Chapingo VF74, to MRMS in Curinda M87 among the dwarfs (**Tables 3** and **4**; **Figures 1** and **2**). The response of the cultivars to the TTKSK race was similar, but the number of resistant were less, and no NI were observed. Among the seedling susceptible (SS), Toluca 53 was resistant; the seedling resistant Crespo 63, Chapingo 53, Yaqui 50, and Yaqui 53 were classified as resistant in the field among the tall cultivars. Saric F70, Guarina 85, and Maya 74 were susceptible at seedling stage, but resistant in the field. In contrast, Kentana 54 among the tall cultivars, Mochis T88 and Yecorato F77 among the dwarfs showed the highest DS against stem rust race TTKSK.

In the case of leaf rust, most of the cultivars varied from near immunity to resistant in both Tall and Dwarf cultivars. Crespo 63, Kentana 52, Yactana Tardio, Frontera, Candeal 48, Yaqui 50, Egypt 101, Kentana 51, Candeal 52, Narinio 59. Bajio53, and Toluca 53 were NI. Among the dwarfs, Mochis T88 and Palmerin F2005 were NI, whereas Tobari 66, Jaral F66, Saric F70, Orizaba 77 Guarina 85, and Tepoca M89 were resistant to leaf rust race MBJ/SP, except for Chapingo VF74 (MRMS).

Yellow rust resistance levels were less than those shown by tall and dwarf cultivars in the response to leaf rust. However, Kentana 52 and Crespo 63 among the tall cultivars were SS but highly resistant, respectively at the adult stage, other seedling resistant cultivars were Narino 59, Chapingo 48, and Kentana 48. Whereas Orizaba 77, Chapingo VF74, Palmerin F2005, Maya S2007, and Huites F95 were classified into the resistant group.

### LTN and PBC

**Figure 3:**
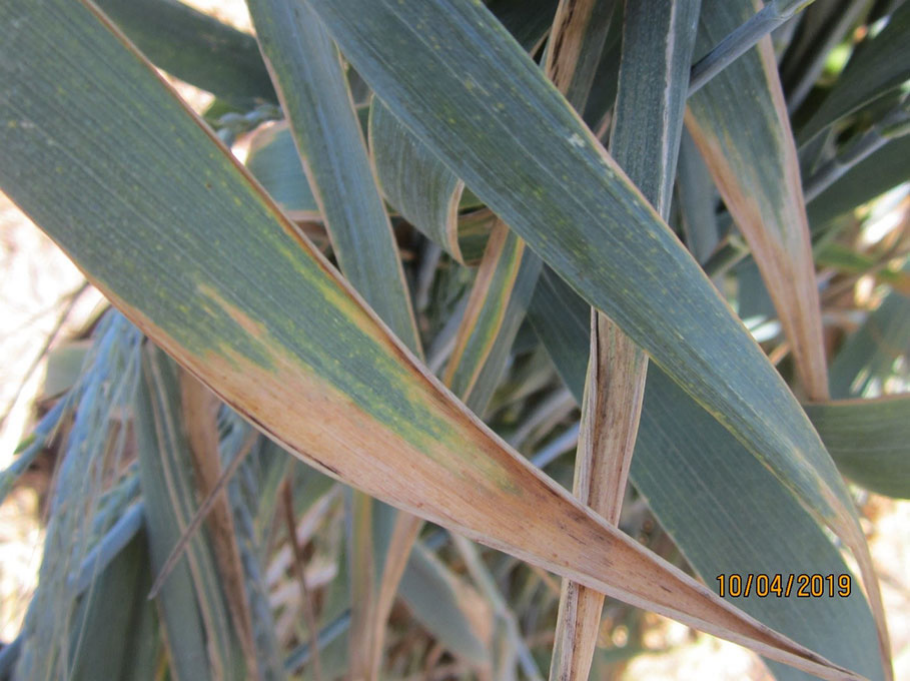
Leaf tip necrosis (LTN) on wheat flag leaves.

**Figure 4:**
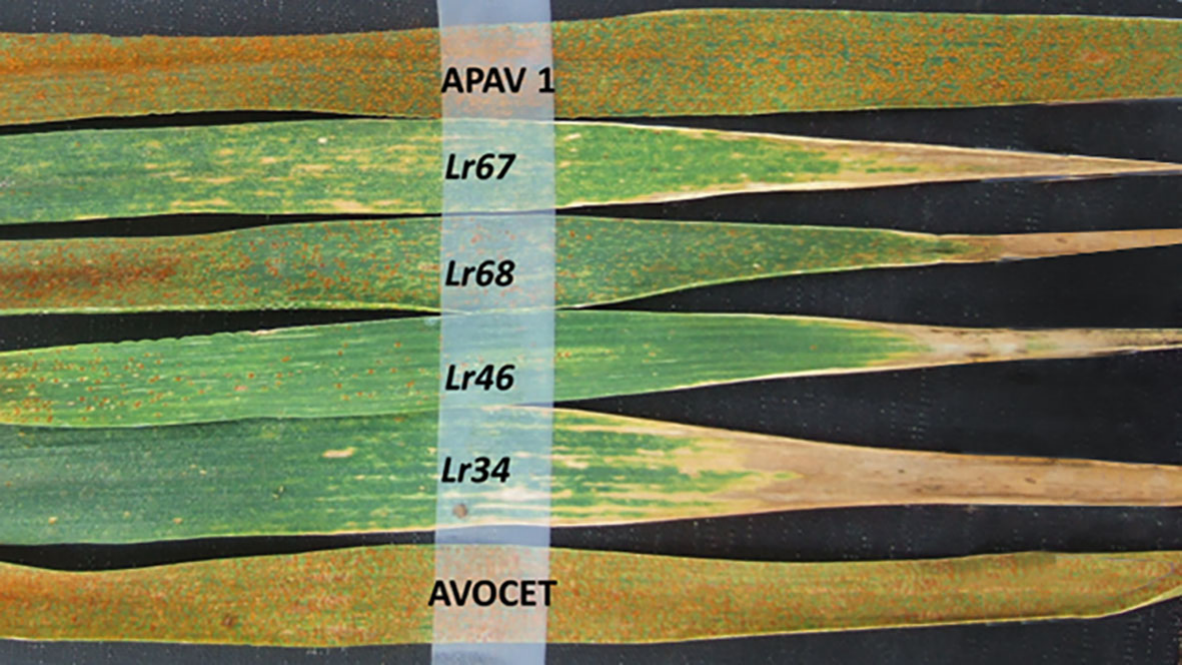
Leaf tip necrosis (LTN) displayed on the flag leaf associated with the leaf rust (LR) resistance genes *Lr34*, *Lr46*, *Lr67* and *Lr68* in Avocet background.

**Figure 5:**
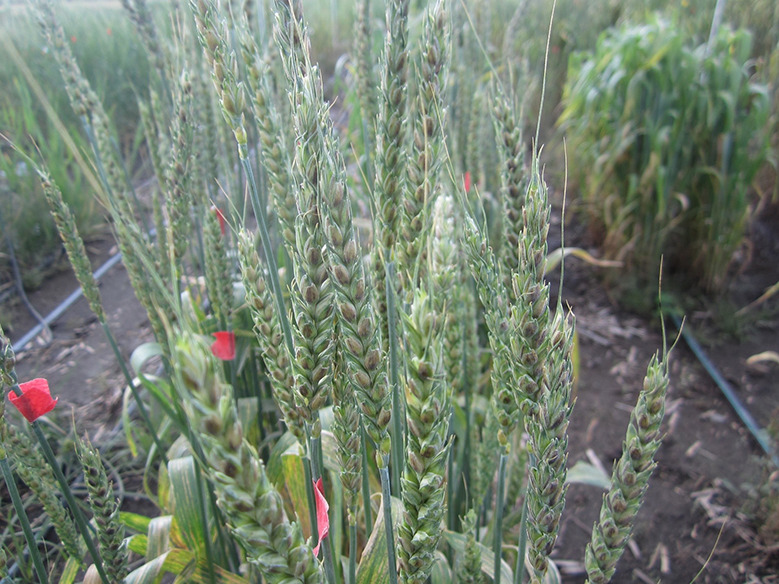
Brown necrosis or Pseudo Black Chaff (PBC) observed on the glumes of wheat heads associated with stem rust (SR) resistance gene *Sr2*.

LTN (**Figures 3** and **4**) was observed in almost all cultivars tested, except for Candeal 48 Candeal 52 and Yaktana Tardio. In contrast, PBC or brown necrosis (**Figure 5**) was not clear or conspicuous in Lerma, Huamatla, Kentana 52, Huamantla Rojo, Yaktana Tardio, and Toluca 53 among the tall cultivars (**Table 5**) or in Orizaba 77, Huites F95, Bajio M67, Yecorato F77, Victoria M81, and Tepoca M89 among the dwarf cultivars (**Table 6**).

**Table 5:** Molecular markers associated with the slow rusting resistance genes *Lr34/Yr18/Sr57/Ltn1, Lr46/Yr29/Sr58/Ltn2, Lr67/Yr46/Sr55/Ltn3, Lr68, Sr2/Yr30*, and the morphological traits leaf tip necrosis (LTN) and pseudo black chaff (PBC) displayed by tall Mexican wheat cultivars.

VARIETY	Year of release	Morphological trait		Molecular Marker				Stem rust	
		LTN	PBC	*Lr34*_TCCIND	*Lr46*_snp	TM4*Lr67*	*Lr68* (SNP)	Lr gene Combination	*Sr2_ger9 3p*
SUPREMO 211	1945	+	+	*Lr34*	*Lr46*			*Lr34+Lr46*	
FRONTERA	1945	+		*Lr34*	*Lr46*		*Lr68*	*Lr34+Lr46+68*	*Sr2*
MARROQUI 588	1945	+	+		*Lr46*	*Lr67*		*Lr46+Lr67*	*Sr2*
CHAPINGO 48	1948	+	+		*Lr46*	*Lr67*		*Lr46+Lr67*	
KENTANA 48	1948	+	+	*Lr34*	*Lr46*			*Lr34+Lr46*	*Sr2*
CANDEAL 48	1948		+		*Lr46*			*Lr46*	*Sr2*
YAQUI 48	1948	+	+		*Lr46*			*Lr46*	
YAQUI 50	1950	+	+		*Lr46*	*Lr67*		*Lr46+Lr67*	
VERANO PELON	1950	+	+	*Lr34*	*Lr46*			*Lr34+Lr46*	
NAYAR	1950	+		*Lr34*	*Lr46*			*Lr34+Lr46*	
CRESPO 63	1963	+	+	*Lr34*	*Lr46*			*Lr34+Lr46*	
LERMA	1950	+		*Lr34*	*Lr46*			*Lr34+Lr46*	
LERMA 50	1950	+	+	*Lr34*	*Lr46*			*Lr34+Lr46*	
LERMA ROJO	1950	+	+	*Lr34*	*Lr46*			*Lr34+Lr46*	
HUAMANTLA	1950	+		*Lr34*	*Lr46*			*Lr34+Lr46*	
EGYPT 101	1950	+	+		*Lr46*		*Lr68*	*Lr46+68*	*Sr2*
KENTANA 51	1951	+		*Lr34*	*Lr46*			*Lr34+Lr46*	
KENTANA 52	1952	+		*Lr34*	*Lr46*			*Lr34+Lr46*	
BAJIO 52	1952	+	+	*Lr34*	*Lr46*			*Lr34+Lr46*	
CANDEAL 52	1952		+		*Lr46*			*Lr46*	*Sr2*
YAKTANA TARD.	1952			*Lr34*				*Lr34*	
YAQUI 53	1953	+	+			*Lr67*		*Lr67*	*Sr2*
CHAPINGO 53	1953	+	+		*Lr46*	*Lr67*		*Lr46+Lr67*	
CONSTITUCION	1953	+	+			*Lr67*		*Lr67*	
TOLUCA 53	1953	+		*Lr34*	*Lr46*			*Lr34+Lr46*	
BAJIO 53	1953	+	+	*Lr34*	*Lr46*			*Lr34+Lr46*	*Sr2*
MAYO 54	1954	+			*Lr46*	*Lr67*		*Lr46+Lr67*	
LERMA ROJO	1954	+	+	*Lr34*	*Lr46*			*Lr34+Lr46*	
HUAMANTLA ROJO	1954	+		*Lr34*	*Lr46*			*Lr34+Lr46*	
KENTANA 54	1954	+	+	*Lr34*	*Lr46*			*Lr34+Lr46*	*Sr2*
NARINO 59	1959	+	+	*Lr34*	*Lr46*			*Lr34+Lr46*	

**Table 6:** Molecular markers associated with the slow rusting resistance genes *Lr34/Yr18/Sr57/Ltn1, Lr46/Yr29/Sr58/Ltn2, Lr67/Yr46/Sr55/Ltn3, Lr68, Sr2/Yr30*, dwarfing genes *Rht-B1* and *Rht-D1*, and the morphological traits leaf tip necrosis (LTN) and pseudo black chaff (PBC) displayed by dwarf Mexican wheat cultivars.

VARIETY	Year of *release*		Morphological Trait			Leaf rust Marker Stem rust				Dwarfing gene	
		LTN	PBC	*Lr46_snp*	*Lr46f2j2*	*Lr68 (SNP)*	*Lr34_TCCIND*	*Combination*	*Sr2_ger93p*	*Rht-B1*	*Rht-D1*
LERMA ROJO 64	1964	+	+	*Lr46*	*Lr46*	**	*Lr34*	*Lr34+Lr46*	**	*Rht-B1*	**
JARAL F66	1966	+	+	*Lr46*	**	**	**	*Lr46*	*Sr2*	**	*Rht-D1*
TOBARI F66	1966	+	+	**	**	*Lr68*	*Lr34*	*Lr34+Lr68*	*Sr2*	**	*Rht-D1*
NORTENO M67	1967	+	+	*Lr46*	*Lr46*	**	*Lr34*	*Lr34+Lr46*	**	*Rht-B1*	**
BAJIO M67	1967	+		**	**	*Lr68*	*Lr34*	*Lr34+Lr68*	*Sr2*	**	*Rht-D1*
SARIC F70	1970	+	+	*Lr46*	**	**	*Lr34*	*Lr34+Lr46*	**	*Rht-B1*	*Rht-D1*
CHAPINGO VF74	1974	+	+	**	**	*Lr68*	*Lr34*	*Lr34+Lr68*	**	**	*Rht-D1*
YECORATO F77	1977	+		*Lr46*	**	**	*Lr34*	*Lr34+Lr46*	**	**	*Rht-D1*
ORIZABA 77	1977	+		*Lr46*	**	*Lr68*	*Lr34*	*Lr34+Lr46+Lr68*	*Sr2*	**	*Rht-D1*
HUASTECO M81	1981	+	+	*Lr46*	**	**	*Lr34*	*Lr34+Lr46*	**	*Rht-B1*	**
VICTORIA M81	1981	+		*Lr46*	**	*Lr68*	*Lr34*	*Lr34+Lr46+Lr68*	*Sr2*	*Rht-B1*	*Rht-D1*
GUARINA 85	1985	+	+	*Lr46*	**	**	**	*Lr46*	**	**	*Rht-D1*
CURINDA M87	1987	+	+	*Lr46*	**	**	**	*Lr46*	*Sr2*	**	**
MOCHIS T88	1988	+		*Lr46*	**	*Lr68*	*Lr34*	*Lr34+Lr46+Lr68*	*Sr2*	**	*Rht-D1*
TEPOCA M89	1989	+		*Lr46*	**	**	*Lr34*	*Lr34+Lr46*	*Sr2*	**	*Rht-D1*
BATAN F92	1992	+	+	*Lr46*	**	**	**	*Lr46*	*Sr2*	**	*Rht-D1*
HUITES F95	1995	+		*Lr46*	**	**	**	*Lr46*	**	*Rht-B1*	**
PALMERIN F2005	2005	+	+	*Lr46*	**	**	*Lr34*	*Lr34+Lr46*	**	**	*Rht-D1*
MAYA S2007	2007	+	+	*Lr46*	**	**	**	*Lr46*	**	**	*Rht-D1*

All dwarf cultivars tested were negative for the Lr67 marker.

### Molecular Marker Detection

All genotypes tested were positive for one or more of the molecular markers associated with the presence of *Lr34/Yr18/Sr57/Ltn1*, *Lr46/Yr29/Sr58/Ltn2*, *Lr67/Yr46/Sr55/Ltn3*, and *Lr68/Ltn4* (**Tables 5** and **6**)

Among the tall genotypes, markers indicative of the presence of *Lr34* and *Lr46* were the most common, followed by the markers associated with *Lr67* and *Lr68* (**Table 5**). Few cultivars showed positive association with a single marker, i.e., *Lr34* alone in Yaktana Tardio; Lr46 alone in Candeal 48, Yaqui 48, Candeal 52, and Bajio 53; *Lr67* alone in Yaqui 50, Chapingo 53 Yaqui 53, and Constitucion. However, no cultivar with the *Lr68* linked marker alone was identified. The gene combinations, *Lr34*+*Lr46* was the most frequent, followed by *Lr46*+*Lr67*. The combination *Lr46*+*Lr68* was only present in Egypt 101 and *Lr34*+*Lr46*+*Lr68* was identified in Frontera. The combinations *Lr34*+*Lr67*, *Lr34*+*Lr68*, and *Lr67*+*Lr68* were not observed among the cultivars tested.

All cultivars positive for *Lr67* were tested several times including RL6077 in order to validate the marker(s) and the methodologies for a new discovered-cloned leaf rust resistance gene.

Among the dwarf cultivars (**Table 6**), the frequency of markers associated with *Lr34* and *Lr46* were the most common, followed by that of *Lr68*. *Lr46* alone was identified in Guarina 85, Huites F95, Maya S2007, Batan F92, and Curinda M87. No cultivars positive for *Lr67* alone or in combination were identified. No *Lr34* or *Lr68* alone were identified. The most common combinations were *Lr34*+*Lr46*, followed by the *Lr34*+*Lr68* in Chapingo VF74, Tobari F66, and Bajio M67. Victoria M81, Orizaba 77, and Mochis T88 were positive for the combination *Lr34*+*Lr46*+*Lr68*.

*Sr2* associated molecular markers were positive in Frontera, Marroqui 588, Kentana 48, Candeal 48, Candeal 52, Yaqui 53, Bajio 53, Kentana 54, and Egiptot 101 among the tall cultivars (**Table 5**). Among the dwarfs, the *Sr2* molecular marker was positively associated in Tobari F66, Jaral F66, Bajio M67, Orizaba 77, Victoria M81 Curinda M87, Mochis T88, Tepoca M89, and Batan F92 (**Table 6**).

Molecular markers associated with the dwarfing genes *Rht-B1* and *Rht-D1* were identified in 18 of the 19 dwarfs. *Rht-D1* was the most common when compared to that of *Rht-B1*. Saric F70 and Victoria M81 carried the *Rht-B1*+*Rht-D1* combination. However, Curinda M87 carries neither *Rht-B1* nor *Rht-D1* (**Table 6**).

## Discussion

The concept of multiple disease resistance in cultivars containing the slow rusting genes and triple rust resistance were conceived by Dr. Borlaug and emphasized in his breeding schemes. Such concepts remain incorporated in the current breeding schemes at CIMMYT in the form of durable resistance genes which are effective against the actual rust races present worldwide. Bread wheat cultivars released in Mexico carried high levels of resistance to stem rust, leaf rust, and yellow rust. The molecular marker analysis revealed that most cultivars carry at least one or more of the following slow rusting resistance genes: *Lr34/Yr18/Sr57*, *Lr46/Yr29/Sr58*, *Lr67/Yr46/Sr55*, *Sr2/Yr30*, and *Lr68*. Among the tall and dwarf cultivars evaluated, there is a considerable variation in their response to rust in the different locations and years. When the presence of a single gene—based on the molecular markers of a determined bread wheat cultivar—showed a higher level of resistance compared to a cultivar with the presence of more than one resistance factor based on the markers, the difference could be attributed to the presence of additional, not yet cataloged slow rusting resistance genes. There exists great variation in cultivar resistance (APR) to the three rusts. In some cases, levels of APR are similar between cultivars independently of whether the cultivars were seedling resistant or susceptible. In fact, as expected, Chapingo 53, Yaqui 53, and Crespo 63, showed similar levels of APR as Supremo 211, Verano Pelon, Toluca 53, and Bajio 53 cultivars which were SS. However, the level of yellow rust resistance might not be adequate as with Verano Pelon, Yaqui 50, and Yaqui 53. The opposite was observed in the response to stem rust RTR and TTKSK races in the cultivars Frontera, Kentana 48, and Lerma 50 in which the levels of resistance to stem rust were not adequate, but high levels of resistance to yellow and leaf rust were displayed.

High levels of resistance to leaf rust exist in all the cultivars tested except for Yaqui 48 among the tall cultivars and Chapingo VF74 among the dwarfs, but response to other rust diseases varied from highly resistant to susceptible. In the case of the yellow rust, cultivars such as Kentana 48 and Kentana 52 were highly resistant but their resistance to stem rust was intermediate.

Resistance in Supremo 211, Yaqui 53, Toluca 53, Bajio 53, Narinio 59, and Crespo 63 could be attributed to the gene combination *Sr57*+*Sr58* and the presence of *Sr2* indicated by brown necrosis. In Yaqui 53, however, the resistance could be attributed to the presence of *Sr55* and *Sr2*. Supremo 211 clearly showed the necrosis in the glumes and internodes as noted by Dr. Borlaug since their release in Mexico in 1945 (Borlaug et al., 1949); however, the *Sr2* marker used was negative in this variety. The same combination among the dwarfs Saric F70 and *Sr58*+*Sr2* in Guarina 85 and Maya S2007 confers high levels of resistance.

In the case of Ug99, Chapingo 48 was positive for *Sr55* and *Sr58* and showed moderately resistance; but Candeal 52 was positive for *Sr2* and *Sr58*. Among the Dwarfs, Tobari 66, Orizaba 77, and Palmerin F2005 were MR to leaf rust, but moderate susceptible to Ug99 stem rust.

Gene combinations in Verano Pelon, Egypt 101, and Yaqui 50, while being effective against stem and leaf rust races, may not be enough against present yellow rust races with more virulence factors. The opposite can be found where gene combinations *Sr57*+*Sr58* in Kentana 51 and Kentana 52, or *Sr57*+*Sr58*+*Sr2* in Kentana 48 are highly effective against leaf and yellow rust, but moderately susceptible to RTR and Ug99 stem rust.

Mayo 54 and Marroqui 588, although resistant to leaf and yellow rust, showed marginal resistance to stem rust RTR and Ug99 races. Constitucion (*Sr55/Lr67/Yr46*), on the other hand, was resistant to stem and leaf rust, but moderately susceptible to Ug99 and yellow rust. Chapingo VF74 and Huites F95 were resistant to yellow and stem rust but moderately resistant to leaf rust and Ug99 stem rust. Other dwarfs, such as Jaral 66, Curinda M87, and Mochis T88 although showing an adequate level of resistance to leaf rust, demonstrated low resistance to stem rust RTR, Ug99 and yellow rust.

It is expected that, as the number of slow rusting resistance alleles increases in the cultivars, the levels of slow rusting resistance should increase (Singh et al., 2000), Near immunity is expected against leaf rust when two to three slow rusting genes are together (Singh et al., 2000). The same is expected when three to four genes are present against yellow rust and more than five in the case of stem rust (Knott, 1988). Although that has proven to be true in most cases, cultivars in our study showed near immunity response to stem rust race while associated with one or two markers as was observed in Candeal 48 and Candeal 52 with *Sr58* or in Yaqui 53 with *Sr2*+*Sr55* and Constitucion with *Sr55*, indicating that other nonidentified slow rusting genes are present in those cultivars. Another example is the combination *Sr2*+*Sr57*+*Sr58* which confers a NI response in Verano Pelon and Supremo 211 which was also found in the cultivars Kentana 54 and Lerma 50; but the different response of these cultivars to stem rust indicates the presence of other resistance genes in Verano Pelon and Supremo 211. One slow rusting gene alone conferring near immunity is unusual or never yet seen; therefore, more nonidentified or not yet cataloged genes must be present.

In the case of TTKSK (Ug99) stem rust, no NI groups were observed. Combination *Sr57*+*Sr58*+*Sr2* in Crespo 63, Toluca 53, and Narinio 59 were grouped in the resistant category, but the same combination in Kentana 48, Lerma 50, and Kentana 54 and others resulted in their being grouped in the moderately susceptible category (up to 60% DS), again indicating the presence of other resistance genes besides those inferred by the molecular markers tested in the study.

In the case of leaf rust, apparently, a single slow rusting gene is enough to reduce the DS to a minimum in the tall cultivars, as in Yaktana Tardio (*Lr34*), Candeal 48 (*Lr46*), and Yaqui 53 (*Lr67*). In other instances, a combination of two resistance genes, as in Kentana 52 (*Lr34*+*Lr46*), Yaqui 50 (*Lr46*+*Lr67*), or Egypt 101(= Kenya Governor) (*Lr46*+*Lr68*) are required, but three are required in the case of Frontera (*Lr34*+*Lr46*+*Lr68*). Among the dwarfs, Palmerin F2005 was highly resistant, but DS in most cases varied from resistant in Jaral F66 (*Lr46*) and Guarina 85 (*Lr46*), to MR in Batan F92 (*Lr46*), Huites F95 (*Lr46*), and Maya S2007 (*Lr46*). The same grouping was observed when a two gene combination existed: in Tobari F66 (*Lr34*+*Lr68*), Saric F70 (*Lr34*+*Lr46*) in the resistant group; and Bajio M67 (*Lr34*+*Lr68*) and Huasteco M81 (*Lr34*+*Lr46*) in the MR group. The gene combination of *Lr34*+*Lr46*+*Lr68*, present in Orizaba 77 and Victoria M81, gave a different rust response.

New races with new virulences and adaptation to warmer temperatures are common in the yellow rust populations. Therefore, a single resistance gene is not going to be enough, as in the case of leaf rust, i.e., Yaktana Tardio (positive for *Yr18*) showed a maximum DS of 40%. Among the cultivars positive for the *Yr29*+*Yr30*, Candeal 48, Candeal 52, Bajio 53, and Yaqui 48 showed different degrees of resistance. Yaqui 53 (*Yr30*+*Yr46*) evidently carries additional alleles in order to be able to reduce the DS. The *Yr18*+*Yr29* combination was very effective in Bajio 53, but less in Nayar, Lerma or Huamantla Rojo. Ten cultivars carried the *Yr18*+*Yr29*+*Yr30* combination, which showed differing degrees of resistance, indicating that Narinio 59, Crespo 63, and Kentana 48 evidently carry additional resistance alleles.

*Yr46* was detected alone, as in Mayo 54, being MR, but the *Yr30*+*Yr46* combination displayed a range of rust responses varying from resistant in Chapingo 48 to MR in Yaqui 50 and Chapingo 53. Marroqui 588 carries the same gene combination but may also be carrying *Yr67* (Li et al., 2009; Xu et al., 2014) which is effective in Mexico at the seedling stage and under field conditions as well.

Among the dwarf cultivars, *Yr29*+*Yr30* keeps the yellow rust severity low in Maya S2007, but the same combination does not provide enough levels of resistance in Curinda M86 and Batan F92. In the cultivar Huites F95, where the presence of *Yr30* was difficult to determine by the presence of brown necrosis or the molecular markers, the low DS could not be explained by the presence of *Yr29* alone.

The *Yr18*+*Yr29*+*Yr30* combination among the dwarfs grouped the cultivars positive for these markers into a range of resistant in Palmerin F2005, to moderately susceptible in Lerma Rojo 64. The level of disease provided by the *Yr18*+*Yr29* combination in Yecorato F77, Tepoca M89, Victoria M81, and Mochis T88, were not the same as in Palmerin F2005, apparently due to the lack of the additional resistance provided by *Yr30*. The presence of *Lr68* in Victoria M81 and Mochis T88 indicates that, under the conditions tested, this gene has no effect on yellow rust. The same could be true when the combination *Yr18*+*Yr30*+*Lr68*, as in Chapingo VF74 and Tobari F66, was compared to Bajio 67 (*Yr18*+*Lr68*). Orizaba 77 was nearly immune to yellow rust with the *Yr18*+*Yr29*+*Lr68* combination, but their resistance is more likely due to the presence of a race-specific resistance gene effective at all growth stages. Recently, a study was carried out (Muleta et al., 2017) to determine if *Lr68* influenced yellow rust; the authors indicated that the presence of the marker could have a disease reducing effect.

The slow rusting APR genes *Lr34/Yr18/Sr57*, *Lr46/Yr29/Sr58*, *Sr2/Yr30*, and *Lr68* were introduced into the Mexican germplasm in the first two cultivars released by Dr. Borlaug obtained by selection from crosses made by McFadden (Borlaug et al., 1949): Supremo 211 (Supresa//Hope/Mediterranean) and Frontera (Fronteira//Hope/Mediterranean), both sharing the same parents (Supresa = Polissu/Alfredo Chaves 6.21 and Fronteira = Polissu/Alfredo Chaves 6.21). The combination *Lr46*+*Lr68*+*Sr2* was introduced through Egypt 101 (= Kenya governor). He also introduced *Lr67/Sr55/Yr46* to the Mexican breeding program through Marroqui 588 (Florence/Aurore) in 1945 from Australia (Borlaug et al., 1949). Marroqui 588 is a cross made in 1922 in Australia and first released in Tunisia in 1925 (Wenholz et al., 1939). An additional source of *Lr34* came to Mexico through Mentana, introduced directly from Italy (Borlaug et al., 1949); it is found in the pedigree of Kentana crosses (Kenya/Mentana). The first crosses made by Dr. Borlaug in Mexico were Marroqui 588/Newthatch (Florence/Aurore//Hope/*3 Thatcher), and Kenya/Mentana in 1945 (Stakman et al., 1967). Using a shortcut of producing two generations per year and shuttle breeding between Chapingo and the Yaqui Valley, by 1949, Yaqui 48, Chapingo 48, Nazas 48, and Kentana 48 were multiplied and released. *Lr67/Yr46/Sr55/Ltn3*, through Marroqui 588, added new sources of resistance to the already in use *Sr2/Yr30* from Hope in Supremo 211 (Supresa//Hope/Med), *Lr34*+*Lr46* in Frontera (Fronteira//Hope/Med) and *Lr34*+*Lr46*+*Lr68* released in Mexico in 1945. Marroqui 588 was crossed with Thatcher and the cross was designated as C5 (Gutierres-Cruz, 1956). C5 was released in Mexico as Chapala. In our study, seed of this variety did not germinate, but DNA extracted from the seed indicated that Chapala was positive for the *Lr67* marker. C5 also appears in the pedigree of Anahuac Barbon, Anahuac Pelon, Constitucion, and Leon 15, cultivars all positive for the *Lr67* marker (data not shown) and was used intensively as a recurrent resistant parent (Gutierres-Cruz, 1956). The presence or absence of a resistance gene in a cultivar is the result of the presence of the gene in the parents; for example in the case of *Lr67*, the absence of the gene among the dwarfs can be explained by its absence in the parents rather than the effect of the dwarfing gene *Rht-D1* located in the same chromosome (4DL), as has been suggested (Moore et al., 2015).

*Lr34/Yr18/Sr57* and *Lr67/Yr46/Sr55* molecular markers are undoubtedly linked to the resistance genes and we are confident that the cultivars mentioned indeed carry the gene(s) as is reflected by their levels of resistance. In contrast, despite molecular markers associated with *Sr2/Yr30* and *Lr46/Yr29/Sr58* not being diagnostic, the presence of brown necrosis and LTN lends support for the inferred presence of *Sr2* and *Lr46*, respectively, particularly when LTN is present, and cultivars are negative for the other diagnostic markers.

*Lr34* was first described in Canada by Dyck (1977) in the cultivar Frontana, *Lr46* in Mexico by Singh et al. (1998) in the variety Pavon F76, and *Lr67* by Dyck and Samborski (1979) in the Pakistani accession PI250413. Because NILs RL6077 and RL6058 showed similar responses to leaf rust, we used them at CIMMYT as sources of *Lr34* until diagnostic markers were developed for *Lr34* that showed otherwise (Kolmer et al., 2008; Krattinger et al., 2009; Lagudah et al., 2009; Spielmeyer et al., 2013). Subsequently, the *Lr67/Yr46* locus, which conferred resistance to leaf rust and yellow rust, was mapped to chromosome 4DL in two independent mapping studies (Hiebert et al., 2010; Herrera-Foessel et al., 2011). Herrera-Foessel et al. (2012) described *Lr68* being present in a cultivar derived from the wheat cultivar Parula. All these adult plant slow rusting resistance genes have been used in the Mexican breeding program led by Dr. Borlaug since the release of Supremo 211 (*Lr34*+*Lr46*), Frontera (*Lr34*+*Lr46*+*Lr68*) in 1945, and Chapingo 48 (*Lr46*+*Lr67*) in 1948 (Borlaug et al., 1949).

*Lr34*, *Lr46*, *Lr67*, and *Lr68* are all associated with a trait expressed in the flag leaf after heading known as LTN (Singh, 1992; Navabi et al., 2005; Rosewarne et al., 2006; Herrera-Foessel et al., 2012; Herrera-Foessel et al., 2014). Wheat cultivars carrying LTN display a longer latency period for infection and fewer, smaller rust pustules when compared to a susceptible cultivar in the field or GH. In our study, all cultivars tested were positive for leaf tip necrosis, indicating that at least one slow rusting resistance gene was present. We did not observe an increased level of LTN as the number of slow rusting resistance genes increased. LTN could be an undesirable trait for some breeders due to the reduction of the photosynthetic area; however, the impact on yield is minimum compared to the protection offered by the slow rusting genes and the impact on rust epidemiology (Singh and Huerta, 1997)

Another important durable APR gene that has provided effective resistance for many years is the *Sr2* gene, which, in combination with other unknown minor genes, is referred to as the *Sr2* complex (Rajaram et al., 1988; Singh et al., 2006). This gene, besides conferring resistance to stem rust, confers resistance to yellow rust (Singh et al., 2000; Singh et al., 2005; Mago et al., 2011b). *Sr2* can be associated or identified by the presence of a morphological trait observed as a result of a black pigmentation called brown necrosis or Pseudo-black chaff which occurs around the glumes and the internodes of the stem after anthesis (McFadden, 1939; Borlaug et al., 1949). It has varying degrees of expression depending on the cultivar and environment (Singh et al., 2008).

McFadden (1937) indicates that brown necrosis was the result of infection by stem rust; but Mishra et al. (2005) suggested that resistance was not always associated with brown necrosis; and Kota et al. (2006) reported that the two traits were inseparable by recombination which was subsequently confirmed by Juliana et al. (2015). The presence of necrosis can be noticed in the absence of the disease. The *Sr2* gene was first introduced into the Mexican germplasm by Dr. Borlaug in the Cultivar Supremo 211 (a Hope-derived cross made by McFadden) (McFadden, 1930) and remains as the backbone of stem rust resistance and is associated with the brown necrosis trait (Borlaug et al., 1949).

The slow rusting genes *Lr34*, *Lr46*, *Lr67*, and *Sr2* can be considered as backbone genes, which when present in combination with other major genes and/or with known or unknown small effect or minor genes (QTLs), have provided effective resistance over the years in wheat improvement (Ellis et al., 2014). *Lr68* can be added to the backbone genes as a component of useful slow rusting genes that are important contributors to durable leaf rust resistance. The findings from the Mexican wheats lend further support to the significance of these backbone slow rusting APR genes, albeit in combination with unknown genes, in developing more durable rust resistance in wheat.

## Data Availability Statement

This article contains previously unpublished data. The name of the repository and accession number(s) are not available.

## Author Contributions

JH-E established the rust evaluation nurseries. JH-E, RS, LC-H, and EL took the phenotypic data and wrote the main manuscript. HV-M, and MR-G provided most of the germplasm and conducted rust evaluations. SD and DB-S extracted the DNA samples and run the molecular markers. EL developed and provided the molecular markers. All authors contributed to the article and approved the submitted version.

## Conflict of Interest

EL was employed by the company CSIRO.

The remaining authors declare that the research was conducted in the absence of any commercial or financial relationships that could be construed as a potential conflict of interest.

## Supplementary Material

The supplementary Material for this article can be found on line at: https://www.frontiersin.org/articles/10.3389/fpls.2020.00824/full#supplementary-material

Table S1 Wheat cultivars, their response to rust at seedling and adult plant in several locations and years. Leaf tip necrosis and pseudo Black Chaff and molecular markers associated to rust resistance and dwarfing genes.

Table S2 Molecular markers used in the study to determine the presence/absence of rust resistance and dwarfing genes.
